# Valorization of Pineapple Peel Waste through Immobilized Crude Bromelain for Enhanced Feed Protein Hydrolysis

**DOI:** 10.1021/acsomega.5c08847

**Published:** 2026-01-21

**Authors:** Chanyakan Skulborisutsuk, Maythee Saisriyoot, Songwut Suramitr, Yodying Yingchutrakul, Chutikarn Butkinaree, Ryuichi Egashira, Lapporn Vayachuta, Panida Prompinit

**Affiliations:** † Interdisciplinary of Sustainable Energy and Resources Engineering, Faculty of Engineering, 562633 Kasetsart University, Bangkok 10900, Thailand; ‡ Department of Chemical Engineering, Faculty of Engineering, 54775 Kasetsart University, Bangkok 10900, Thailand; § Department of Chemistry, Faculty of Science, 200230 Kasetsart University, Bangkok 10900, Thailand; ∥ National Center for Genetic Engineering and Biotechnology, 67960 National Science and Technology Development Agency (NSTDA), Pathum Thani 12120, Thailand; ⊥ Department of Transdisciplinary Science and Engineering, School of Environment and Society, Institute of Science Tokyo, Tokyo 152-8550, Japan; # National Nanotechnology Center (NANOTEC), 61191 National Science and Technology Development Agency (NSTDA), Pathum Thani 12120, Thailand

## Abstract

Pineapple processing generates considerable waste with byproducts (peels, cores, crowns, and leaves) often containing high levels of bromelain, a proteolytic enzyme with promising applications in animal feed. However, the instability and low activity of crude bromelain (CBr) in aqueous form limit its industrial utilization. In this study, CBr was extracted from pineapple peel waste and directly immobilized onto a bentonite (Bt)–carboxymethylcellulose (CMC) composite without further purification. The CBr–Bt–CMC composites were prepared via ionotropic gelation, with cysteine incorporated to enhance enzymatic performance. The composite with a bromelain-to-cysteine mass ratio of 1:65 could enhance catalytic activity by over 1,000% compared to the control without cysteine. It exhibited an immobilization yield exceeding 80% with significantly improved thermal stability, retaining nearly twice the activity of free CBr after 10 min at 100 °C. Application of the immobilized CBr in soybean meal (SBM) hydrolysis demonstrated significant improvements in nutritional value (approximately 3-fold), degradation of allergenic proteins, and generation of low-molecular-weight peptides at 60–70 °C within 30 min. Immobilized CBr exhibited sustained catalytic performance, retaining over 60% of its initial activity after four cycles. Collectively, these results highlight the potential of the immobilized crude bromelain as a robust and efficient biocatalyst for the enhancement of nutritional value in feed protein.

## Graphical abstract

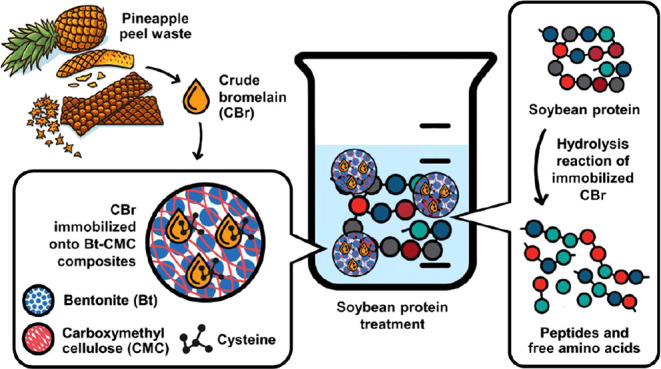

## 1 Introduction

Pineapple (*Ananas comosus*) is extensively cultivated across tropical regions, particularly in South and Central America and Southeast Asia.^1^ Recent market data indicate that worldwide pineapple production amounted to approximately 29.6 million metric tonnes in 2023.^2^ During processing, a large fraction of the fruit is discarded, as only about 60% is consumed, while peels, cores, and stems account for roughly 40–60% of the total biomass.^3^ The accumulation of these residues generates considerable agro-industrial waste, leading to environmental concerns and additional economic burdens associated with disposal.^4^ Despite these challenges, pineapple processing byproducts contain substantial levels of nutrients and bioactive constituents, underscoring their potential for value-added utilization.

Bromelain is the principal enzymatic component recovered from pineapple processing residues and consists of a group of proteolytic enzymes. It occurs throughout the pineapple plant, although its distribution varies by tissue type. Stem tissues primarily contain stem bromelain (EC 3.4.22.32), whereas fruit tissues are dominated by fruit bromelain (EC 3.4.22.33), which is responsible for proteolytic activity in the edible portion of the fruit.^5,6^ Pineapple processing byproducts, particularly peels, typically contain bromelain protein at levels of approximately 5–7%.^7,8^ Functionally, bromelain belongs to the cysteine protease family, as its catalytic mechanism depends on a cysteine residue at the active site. Owing to its strong proteolytic capability, bromelain has attracted attention as an exogenous enzyme for improving the utilization of protein-rich feed materials, including fish meal and soybean meal (SBM). Previous studies have demonstrated that crude bromelain derived from pineapple waste can enhance protein digestibility in SBM and shrimp feed.^9^ In addition, bromelain treatment has been shown to reduce allergenic protein fractions in SBM, contributing to improved growth performance and feed efficiency in broiler production.^10^ Despite these advantages, the direct application of crude bromelain in aqueous form remains challenging due to handling difficulties, interference from coextracted substances and endogenous inhibitors, and limited stability under fluctuating pH and temperature conditions. Consequently, strategies aimed at enhancing the catalytic performance and stability of crude bromelain, while preserving practical applicability, are essential for its expanded use in animal feed and related biotechnological applications.

Immobilization has been widely adopted as an effective approach to overcome the practical limitations associated with free enzymes. This strategy relies on fixing enzymes onto or within solid matrices, which can improve structural integrity and increase tolerance to external stresses.^11^ In addition to facilitating enzyme recovery and reuse, immobilization often enhances operational stability by reducing sensitivity to changes in temperature, pH, solvents, and mechanical forces. As a result, immobilized enzymes are increasingly regarded as suitable candidates for cost-effective, sustainable, and scalable bioprocesses in food- and cosmetic-related applications.^12–14^ In addition to facilitating enzyme recovery and reuse, immobilization often enhances operational stability by reducing sensitivity to changes in temperature, pH, solvents, and mechanical forces. As a result, immobilized enzymes are increasingly regarded as suitable candidates for cost-effective, sustainable, and scalable bioprocesses in food- and cosmetic-related applications.^15–21^ Among these techniques, ionotropic gelation is a simple and mild method that employs biocompatible and nontoxic cross-linking agents and shows high potential for enhancing enzyme stability in animal feed application.^22,23^ In our previous study, we demonstrated that purified stem bromelain immobilized onto a bentonite (Bt)–carboxymethylcellulose (CMC) composite, in the presence of calcium ions using an ionotropic gelation method, significantly improved thermal stability. This system achieved an immobilization yield greater than 95%, with minimal enzyme leakage in aqueous media.^24^ Moreover, the immobilized enzyme enhanced the nutritional value of SBM by approximately 4-fold and effectively reduced antinutritional factors (ANFs) at 60 °C within 30 min. Despite these advances, the immobilization of crude bromelain directly derived from pineapple peel waste remains unexplored.

Recent reviews have described diverse bromelain immobilization strategies, largely based on purified enzymes and conventional polymeric or inorganic supports.^25^ In contrast, this study employs a feed-grade bentonite (Bt)–carboxymethylcellulose (CMC) composite formed via ionotropic gelation to directly immobilize crude bromelain (CBr) extracted from pineapple peel waste. CBr is obtained via cold aqueous extraction followed by centrifugation. The extracted enzyme is subsequently immobilized onto Bt–CMC support in the presence of an enzymatic activator to produce CBr–Bt–CMC composite. Carboxymethylcellulose (CMC) consists of cellulose backbones substituted with carboxymethyl and hydroxyl groups, imparting water solubility and an overall negative charge.^26^ Bentonite (Bt), a widely available and inexpensive clay material, is primarily composed of montmorillonite layers that possess permanent negative charges arising from structural charge imbalance. Its layered structure confers a notable cation-exchange property. The anionic character of the CMC–bentonite matrix promotes electrostatic association with positively charged amine groups of bromelain, mediated by calcium ions supplied by CaCl_2_, which functions as an economically viable and biocompatible cross-linking agent.^27^ The incorporation of enzymatic activators such as cysteine,^28–30^ calcium ion,^28,31^ and sucrose^32,33^ has been widely investigated to enhance enzyme activity. Among these, cysteine, a thiol-containing amino acid, can directly participate in the catalytic process of bromelain. It has shown superior efficacy in promoting bromelain activity even at low concentrations compared to other activators.^30^ Therefore, cysteine is selected as the preferred activator in this study, and various bromelain-to-cysteine mass ratios are tested during the immobilization process. This integrated design differentiates the present system from previously reported bromelain immobilization approaches and underscores its suitability for high-temperature protein hydrolysis and sustainable feed applications. Key parameters of the immobilization process, enzymatic activity, and structural characteristics of the resulting composites are systematically investigated. Thermal stability and catalytic efficiency of immobilized CBr are evaluated and compared to both free CBr and immobilized CBr without cysteine. Additionally, hydrolytic performance of the composites is assessed in treatment of soybean meal (SBM). A schematic overview of CBr immobilization process onto Bt–CMC composites and their application in soybean protein hydrolysis is illustrated in Figure 1. This work contributes to green chemistry and the circular bioeconomy by converting pineapple peel waste into a value-added immobilized bromelain system using low-energy, nontoxic materials, thereby enabling sustainable enzyme stabilization and improved protein utilization for feed applications.

**Figure 1:**
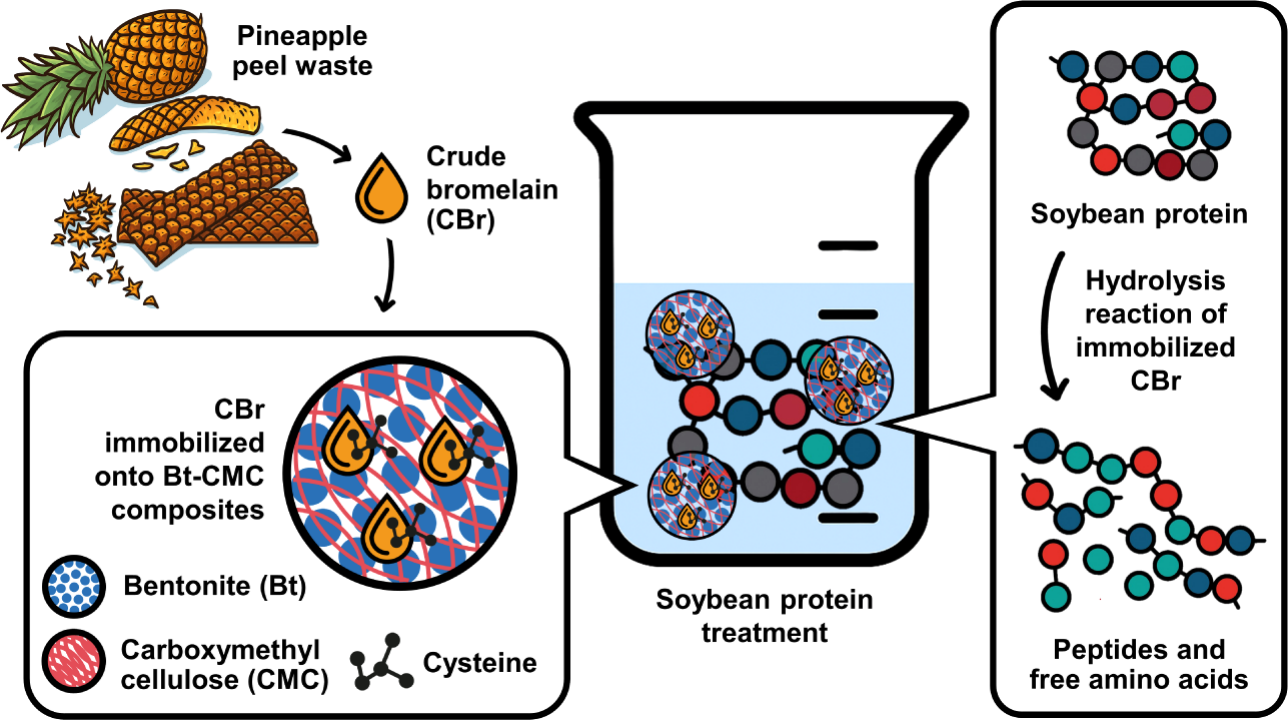
Conceptual illustration of Bt–CMC composite formation with immobilized crude bromelain and its application in soybean protein hydrolysis.

## 2 Materials and Methods

### 2.1 Materials

Pineapple peels from the Smooth Cayenne (*Ananas comosus* L.) were obtained from a local fresh fruit stall in Khlong Luang district of Pathum Thani province, Thailand. They were stored at a freezing temperature below −20 °C until used to sustain the enzymatic activity of bromelain. Sodium bentonite (Bt) clay was purchased from a local supplier under the brand name of Trubond (Sibelco Australia). Sodium carboxymethyl cellulose (sodium CMC#2000, food grade) was purchased from UNION CHEMICAL 1986 Co., Ltd. (Thailand). Calcium chloride dihydrate (food additive, 74%) was purchased from KRUNGTHEP CHEMI Co., Ltd. (Thailand). L-cysteine hydrochloride monohydrate (analytical grade, 99%) was sourced from Thermo Fisher Scientific (Waltham, MA, USA). Ninhydrin (1,2,3-indantrione monohydrate; ACS grade, ≥98.0% by UV analysis) was supplied by Fluka, and glycine (purity >99.0%) was supplied by Tokyo Chemical Industry Co., Ltd. Absolute ethanol (≥99.9%) was acquired from Merck. Soybean meal used in this study was purchased from a local market in Bangkok, Thailand. All reagents and experimental solutions were prepared using deionized water with a resistivity of 18.2 MΩ·cm.

### 2.2 Crude Bromelain Extraction

Pineapple peels were first cut into small fragments and homogenized with chilled deionized water at a 1:1 (w/w) ratio for 2 min. The homogenate was passed through cheesecloth to remove solids, followed by centrifugation at 10,000 × g for 20 min at 4 °C. The resulting supernatant, designated as crude bromelain (CBr), was recovered and stored below −20 °C until further use.

### 2.3 Immobilization of Crude Bromelain onto Bentonite-Carboxymethylcellulose Composites

The CBr extracted from pineapple peel was immobilized onto Bt–CMC composites by ionotropic gelation technique. First, cysteine was added to CBr and stirred until fully dissolved. A suspension of Bt/CMC in CBr was then prepared by stirring for 15 min. Subsequently, an aqueous calcium chloride solution was added, and the mixture was further stirred for 30 min to produce a mixture in which Bt, CMC, and Ca^2+^ were present at relative masses of 20:1:6. The composites were prepared in different bromelain-to-cysteine mass ratios of 1:0, 1:20, 1:35, 1:50, 1:65, 1:80, and 1:95. The resulting filtrate was collected and analyzed for soluble protein content. The resulting solids were recovered by vacuum filtration, dried at 60 °C for approximately 12 h, ground, sieved to particle sizes below 250 μm, and kept below −20 °C.

### 2.4 Determination of Protein Content and Enzymatic Performance

The concentration of soluble protein in CBr and other samples was quantified using a Bradford-based assay, with bovine serum albumin (BSA) employed as the calibration standard.^22^ The catalytic performance of CBr and related samples was evaluated using a casein hydrolysis assay adapted from previous reports,^22^ with 0.65% (w/v) bovine milk casein as the substrate. Reactions were conducted at 37 °C for 10 min, after which absorbance of the supernatants was measured at 660 nm using a microplate spectrophotometer (BioTek, PowerWave XS2). Enzyme performance was reported as units of catalytic output (U/mg solid Br and U/mg sample), corresponding to the liberation of 1 μmol of tyrosine per minute under the specified conditions.

### 2.5 Evaluation of Immobilization Efficiency and Enzymatic Performance

Evaluation of enzyme loading efficiency and biocatalytic function of crude bromelain (CBr) on the Bt–CMC composite were determined based on the reduction in total protein concentration in the immobilization supernatant. Protein concentrations before and after immobilization were quantified using a standard protein assay. The yield of enzyme attachment was determined based on the relative reduction in soluble protein compared to the starting concentration. As the enzyme employed in this study was crude bromelain extracted from pineapple peel waste, the immobilization yield represents the overall retention of proteolytic protein fractions rather than selective immobilization of purified bromelain. This approach was adopted to evaluate the practical efficiency of immobilizing a crude enzyme system intended for feed applications. The functional relevance of the immobilized system was subsequently confirmed through enzymatic activity assays, thermal stability measurements, and soybean meal hydrolysis performance. It should be noted that activity-based immobilization metrics may provide additional mechanistic insight and are considered an important subject for future investigation. Following composite formation, bromelain coupling was quantified in terms of loading capacity (LC%) and immobilization yield (IY%). Protein concentrations in the initial CBr solution and in the resulting filtrate were first quantified using the Bradford assay. LC% was calculated as the ratio of bromelain incorporated into the composite to the total mass of Bt, CMC, and cysteine used for composite preparation (*M*_Bt+CMC+cysteine_), as defined in eq 1).^22^ The IY% was determined from the relative decrease in bromelain concentration between the initial solution (Br_initial_)­and the filtrate (Br_filtrate_), according to eq 2). Enzymatic performance was expressed in activity units (EA), defined as the amount of enzyme catalyzing the formation of 1 μmol of tyrosine per minute.^2,2^ In this study, EA was normalized per milligram of solid bromelain or per milligram of sample, as described in eq 3),

1 $$LC\%=\left(\frac{Br_{initial}-Br_{filtrate}}{M_{Bt+CMC+cysteine}}\right)\times 100$$

2 $$IY\%=\left(\frac{Br_{initial}-Br_{filtrate}}{Br_{initial}}\right)\times 100$$

3 $$EA\left(U\right)=\frac{T_{released}\times V_{total}}{M_{enzyme}\times V_{UV}\times t}$$

where *T*_released_ represents the micromoles of tyrosine equivalents released, *V*_total_ is the total assay volume (mL), *M*_enzyme_ is the mass of enzyme or enzyme-containing sample (mg), *V*_UV_ is the volume used for colorimetric analysis (mL), and *t* is the reaction time (min),

### 2.6 Structural and Physicochemical Analysis of Composites

The chemical functionalities of the composites were examined using Fourier transform infrared (FT-IR) spectroscopy with an attenuated total reflectance (ATR) accessory (Thermo Scientific Nicolet iS50). Spectra were collected over the range of 400–4000 cm^–1^ at a resolution of 4 cm^–1^, averaging 64 scans per sample. Crystalline structure was analyzed by X-ray diffraction (XRD) using a Bruker D8 Advance diffractometer equipped with a Cu radiation source (λ = 0.15418 nm), operated at 40 kV and 40 mA. Diffraction patterns were recorded over a 2*θ* range of 4°–25°, with a step size of 0.02° and a counting time of 2 s per step. The basal spacing (*d*_001_) of bentonite was calculated using Bragg’s law (eq 4),^34^

4 nλ=2dsin⁡θ

where *n* is the diffraction order, λ is the incident wavelength, *d* represents the interplanar spacing, and θ denotes the diffraction angle.

### 2.7 Thermal Resistance of Free and Support-Bound Crude Enzyme

Thermal resistance was assessed by incubating free and support-bound CBr in 50 mM phosphate buffer (pH 7.0) at 25 and 100 °C under substrate-free conditions. Following a 10 min heat treatment, samples were allowed to equilibrate to ambient temperature prior to subsequent catalytic activity measurements.

### 2.8 Effect of pH on the Activity of Free and Support-Bound Crude Enzyme

The optimum pH of free and support-bound crude bromelain was evaluated by incubating the samples with 0.65% (w/v) casein solutions prepared in buffers of varying pH at 37 °C. Phosphate buffer solutions (50 mM, pH 3.0–8.0) were prepared using 1 M K_2_HPO_4_ and 1 M KH_2_PO_4_, while Tris–HCl buffers (50 mM, pH 9.0–10.0) were prepared from 1 M Tris base and 1 M HCl. Enzymatic performance was determined using the assay procedure described above.

### 2.9 Stability under Storage Conditions

Stability of immobilized crude enzyme under storage conditions was evaluated over a 10-day period. Samples were stored in airtight containers at 4 °C and 25 °C. In addition, a preliminary assessment was conducted by storing immobilized CBr at 25 °C in vacuum-sealed bags to minimize exposure to air and moisture. Enzymatic performance was measured daily, and residual activity was calculated by normalizing the activity measured on day 1 to 100%.

### 2.10 Performance Evaluation of Immobilized Crude Bromelain in Soybean Meal Hydrolysis

Performance of the immobilized crude bromelain was evaluated by considering hydrolysis of soluble protein in soybean meal (SBM). This method was adapted from a prior study.^35^ Briefly, soybean meal powder (<250 μm) was mixed with immobilized CBr at a mass ratio of 1:4.5, followed by the addition of 6 mL of deionized water. For performance evaluation, soybean meal hydrolysis was conducted at 50, 60, 70, and 80 °C for incubation times of 15 and 30 min in a shaking water bath operated at 120 rpm. Reusability of the immobilized crude bromelain (CBr) was assessed through successive hydrolysis cycles performed at 60 °C for 15 and 30 min under the same agitation conditions. Following incubation, the reaction mixture was heated at 95 °C for 30 min to terminate enzymatic activity, after which 4 mL of deionized water was added. The suspension was centrifuged at 8000 g for 10 min at 4 °C, and the resulting supernatant was recovered for determination of free alpha-amino nitrogen (FAN) using the ninhydrin assay.^36^ Briefly, 1 mL of supernatant was diluted with 3 mL of deionized water and reacted with 1 mL of 2% (w/v) ninhydrin solution prepared in ethanol. The mixture was vortexed and heated at 95 °C for 15 min, followed by cooling for 20 min. Subsequently, 1 mL of 50% (v/v) ethanol was added, and absorbance was measured at 570 nm. FAN concentration was calculated using a glycine calibration curve according to Equation (eq 5),

5 $$\mathrm{FAN}\left(\mathrm{mg}/L\right)=\frac{A_{S}-A_{B}}{A_{G}-A_{B}}\times 2\times F$$

where *A*_S_ represents the mean absorbance of the sample, *A*_B_ denotes the mean absorbance of the blank, and *A*_G_ corresponds to the mean absorbance of the glycine standard solution. Value 2 refers to the concentration of the glycine standard (mg/L), and *F* is the sample dilution factor. Released FAN level of an individual component, composite (FAN_composite_), SBM (FAN_SBM_), and the mixture between the composite and SBM after treatment period (FAN_composite & SBM_), was predetermined for calculation. FAN product derived from SBM hydrolysis (FAN_product_) by the immobilized enzyme was calculated using Equation (eq 6).

6 $$FAN_{product}\left(\mathrm{mg}/L\right)=FAN_{composite\;\&\;SBM}-FAN_{composite}-FAN_{SBM}$$

### 2.11 Sample Preparation and SDS–PAGE Analysis

SDS–PAGE analysis was performed using untreated soybean meal (SBM), the CBr–Bt–CMC composite, and SBM hydrolyzed by the composite at 60 and 70 °C for 30 min. For sample preparation, 0.1 g of each material was suspended in 6 mL of deionized water and heated at 95 °C for 30 min, followed by the addition of 4 mL of deionized water. The suspensions were centrifuged at 8000 g for 10 min at 25 °C, and the resulting supernatants were recovered for removal of interfering substances prior to electrophoretic analysis. Desalting was carried out by loading 500 μL of each supernatant onto a 5 mL HiTrap desalting column (Cytiva), with elution performed using two column volumes. An aliquot of 15 μL of each desalted sample was mixed with NuPAGE LDS sample buffer (Invitrogen, USA) and heated at 70 °C for 10 min. The prepared samples were resolved on 12% SDS–PAGE gels, alongside 2 μL of a prestained protein molecular weight marker (PageRuler, Thermo Scientific, Lithuania). Electrophoresis was conducted using a mini-gel system (Invitrogen, USA) at a constant voltage of 120 V for 90 min. Gels were subsequently stained with colloidal Coomassie Brilliant Blue G-250 at 4 °C, followed by destaining with type I Milli-Q water until clear protein band patterns were obtained. Molecular weight distributions and relative band intensities were then analyzed.

## 3 Results and Discussion

### 3.1 Immobilization of Crude Bromelain onto Bentonite-Carboxymethylcellulose Composites

The effect of cysteine on CBr immobilization was assessed by analyzing LC%, IY%, and EA across various bromelain-to-cysteine mass ratios of 1:0, 1:20, 1:35, 1:50, 1:65, 1:80, and 1:95. Figure 2(a) illustrates LC values of all samples in the range of 0.02–0.07%. Among them, the 1:50 sample showed the highest LC% (0.07 ± 0.006%), while the 1:65 sample provided the lowest LC% (0.02 ± 0.005%). Immobilization yield (IY%) was further analyzed and all samples showed an average IY% higher than 80% as presented in Figure 2(b). EA was further analyzed from two perspectives: EA expressed in units/mg of solid bromelain (Figure 2(c)) and EA expressed in units/mg of sample (Figure 2(d)). Across all formulations, EA values ranged from 1.78 to 60.03 U/mg of solid Br and from 0.001 to 0.014 U/mg of sample. The highest EA in the unit per milligram of solid bromelain was observed from 1:65 sample (60.03 ± 3.50 U/mg of solid Br). These EA values were significantly improved by 3,281% compared to the sample prepared without cysteine (1:0 sample). The 1:65 sample also provided a high EA value of 0.013 ± 0.001 U/mg of sample, which was 1,140% more than that of the 1:0 sample (without cysteine). EA enhancement is likely caused by conformational changes of bromelain, which contains sulfhydryl group at its active center, in response to the presence of cysteine. The findings are consistent with those reported in previous studies.^28–31^ This configuration facilitated substrate access to the enzyme active sites.^37^ Accordingly, CBr was effectively incorporated into Bt–CMC composites via ionotropic gelation, achieving an immobilization yield exceeding 80%. The inclusion of cysteine markedly enhanced the catalytic performance of the immobilized enzyme. Based on these results, the composite prepared at a bromelain-to-cysteine mass ratio of 1:65 was selected for subsequent thermal stability evaluation and soybean meal hydrolysis.

**Figure 2:**
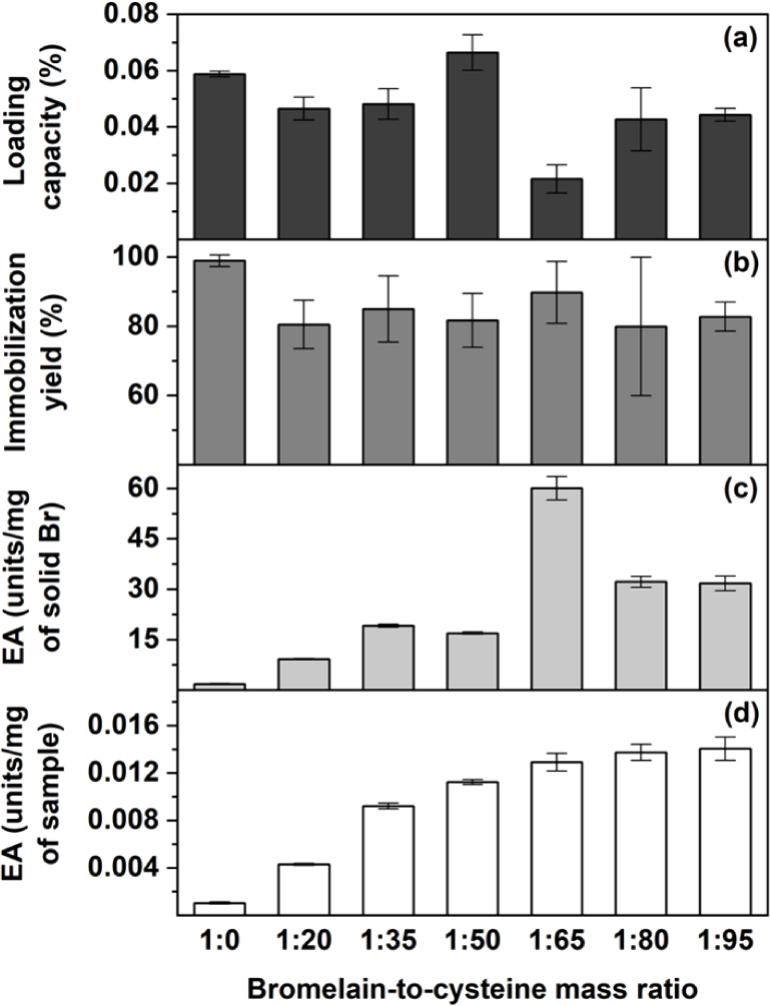
Immobilization efficiency and enzymatic performance of the composites, presented as loading capacity (LC%) (a), immobilization yield (IY%) (b), and enzyme activity expressed in units/mg of solid bromelain (c), and in units/mg of sample (d), for immobilized bromelain prepared at different bromelain-to-cysteine mass ratios (1:0, 1:20, 1:35, 1:50, 1:65, 1:80, and 1:95).

### 3.2 Structural and Physicochemical Analysis of Composites CBr–Bt–CMC Composites

The chemical functionalities of CBr–Bt–CMC composites prepared at bromelain-to-cysteine mass ratios ranging from 1:0 to 1:95 were examined by FT-IR spectroscopy in two spectral regions, namely 400–2000 cm^–1^ (Figure 3) and 2800–4000 cm^–1^ (Figure 4). Prior to composite analysis, the spectra of the individual raw materials—bentonite, CMC, calcium chloride, and cysteine—were recorded for reference. As shown in Figure 3(a), bentonite exhibited characteristic absorption bands at 451, 519, and 1004 cm^–1^, corresponding to Si–O–Si, Al–O–Si, and Si–O stretching vibrations, respectively, along with additional features at 796, 915, and 1633 cm^–1^ associated with Al–Mg–OH stretching, Al–Al–OH stretching, and H–O–H bending.^38^ The spectrum of CMC (Figure 3(b)) revealed peaks at 1018 cm^–1^, 1412 cm^–1^, and 1586 cm^–1^, attributed to C–O–C stretching, CH_2_ bending, and COO- stretching, respectively.^39^ Calcium chloride (Figure 3(c)) exhibited a peak at 1627 cm^–1^ which was assigned to H–O–H bending.^40^ Cysteine (Cys) (Figure 3(d)) showed peaks at 631 cm^–1^ and 737 cm^–1^, contributed to C–S bonds.^41^ Peaks at 1205 cm^–1^ and 1738 cm^–1^ were C–O and C=O stretching, respectively.^42^ Another peak at 1515 cm^–1^ was signified as COO stretching.^43^ The spectra of the immobilized composites (Figure 3(e–k)) were largely governed by the characteristic signals of bentonite, reflecting its dominant contribution to infrared absorption. A weak band near 1423 cm^–1^, corresponding to CH_2_ vibrations of CMC, was also observed. Increasing cysteine content did not result in noticeable spectral changes, likely due to its relatively low proportion within the composite matrix.

**Figure 3:**
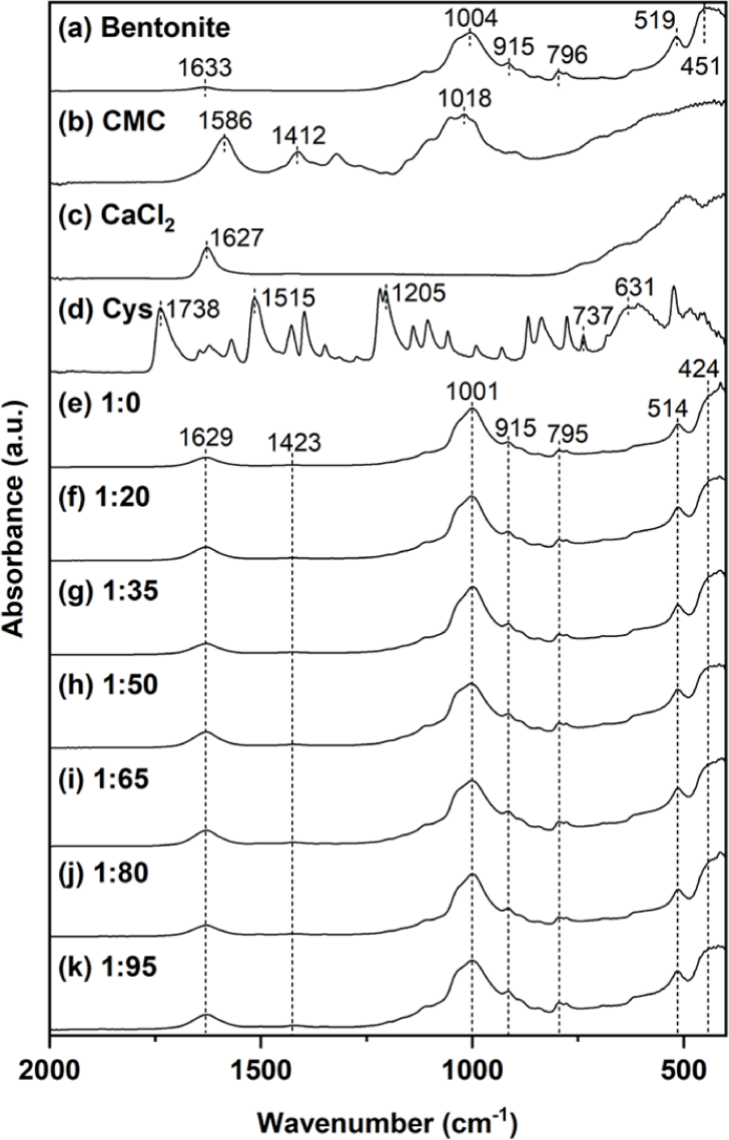
FT-IR spectra recorded in a 400–2000 cm^–1^ region for bentonite (a), CMC (b), calcium chloride (c), cysteine (Cys) (d), and CBr–Bt–CMC composites prepared at bromelain-to-cysteine mass ratios of 1:0 (e), 1:20 (f), 1:35 (g), 1:50 (h), 1:65 (i), 1:80 (j), and 1:95 (k).

**Figure 4:**
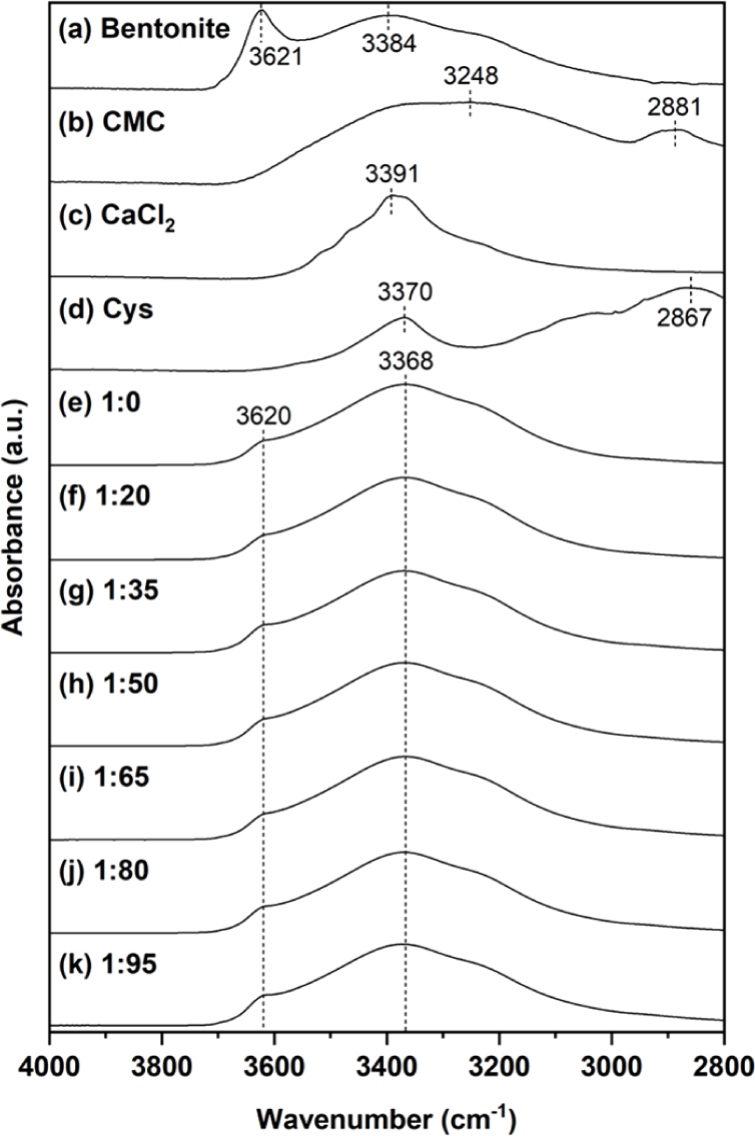
FT-IR spectra recorded in a 2800–4000 cm^–1^ region for bentonite (a), CMC (b), calcium chloride (c), cysteine (Cys) (d), and CBr–Bt–CMC composites prepared at bromelain-to-cysteine mass ratios of 1:0 (e), 1:20 (f), 1:35 (g), 1:50 (h), 1:65 (i), 1:80 (j), and 1:95 (k).

The high-wavenumber region (2800–4000 cm^–1^) of the FT-IR spectra was further investigated. The bentonite spectrum (Figure 4(a)) exhibited a broad absorption band centered at 3384 cm^–1^, attributed to adsorbed water molecules, along with a sharper peak at 3621 cm^–1^ corresponding to structural O–H stretching vibrations.^38^ The CMC spectrum (Figure 4(b)) displayed a band at 2881 cm^–1^, characteristic of C–H stretching vibrations, along with a broad absorption centered around 3248 cm^–1^ attributed to O–H stretching.^39^ Calcium chloride spectrum (Figure 4(c)) exhibited a peak at 3391 cm^–1^ which was assigned to O–H stretching. The spectra of cysteine (Cys) (Figure 4(d)) displayed two peaks including 2867 cm^–1^ and 3370 cm^–1^, attributed to CH_2_ stretching^43^ and N–H stretching vibrations,^42^ respectively. After immobilization, all composites showed similar spectra (Figure 4(e-k)) with main characteristic bands of bentonite. Notably, the bentonite peak at 3384 cm^–1^ was shifted to a lower wavenumber at 3368 cm^–1^. This shift is likely due to overlapping peaks associated with OH groups in CMC, N–H groups in cysteine, and water molecules that interacted with calcium ions.

XRD patterns of immobilized CBr onto Bt–CMC composites with bromelain-to-cysteine mass ratios of 1:0, 1:20, 1:35, 1:50, 1:65, 1:80, and 1:95 were analyzed in a 2θ range of 4°–25° (Figure 5A). Figure 5A­(a) illustrates that bentonite exhibits peaks at 2θ = 5.93° and 19.99° representing clear crystallization, dominated by montmorillonite structure.^44^ These peaks also suggested sodium-rich smectite as the dominant clay mineral, identifying the raw material as sodium bentonite. Observed peaks at 21.04° and 22.18° are attributed to impurities, namely cristobalite and quartz.^44–46^ The XRD profile of CMC (Figure 5A­(b)) exhibited a broad diffraction peak centered at 2θ = 19.93°, indicative of its predominantly amorphous structure with the presence of minor crystalline domains within the cellulose matrix.^47^ Figure 5A­(c) demonstrates a distinctive peak of calcium chloride at 20.57°.^40^ Characteristic peaks of cysteine (Cys) (Figure 5A­(d)) are observed at 18.32°, 21.00°, and 24.54°.^48^ After CBr immobilization, XRD patterns of the composites (Figure 5A­(e-k)) were primarily governed by montmorillonite features. Changes in the interlayer structure of the clay following enzyme incorporation, both in the absence and presence of cysteine, were evaluated by monitoring the basal spacing *d*_001_. This parameter was determined from the 001-diffraction peak located at 2θ ≈ 5°–6° using Bragg’s law (eq 4)). By accounting for the thickness of the silicate layer (∼0.96 nm), the corresponding interlayer distances were calculated and plotted as a function of increasing cysteine mass ratio relative to pristine bentonite (Figure 5B). Accordingly, the interlayer spacings of all composites fell within the range of 0.494–0.566 nm. Pristine Na-bentonite exhibited an interlayer distance of 0.530 nm, attributable to the hydration shells of Na^+^ ions in the bilayer (2W) hydration state.^49^ Following composite formation in the absence of cysteine (1:0), the interlayer spacing decreased markedly to 0.494 nm, which is consistent with the substitution of hydrated sodium ions by hydrated calcium ions within the bentonite galleries during the immobilization process.^35^ However, the interlayer distances showed an increasing trend in a range of 0.535–0.566 nm after addition of cysteine into composites with Br: Cys mass ratios from 1:20 to 1:95. The interlayer spacing of about 0.53 nm implies the occurrence of intercalation of cysteine molecules into bentonite interlayer. Cysteine molecules could lay flat in the interlayer with binding of amino groups onto acidic sites of bentonite and leaving thiols of the cysteine unbound.^50–52^ This free sulfhydryl of the cysteine might play a role in promoting the activity of the immobilized bromelain by breaking the disulfide bonds presented in the bromelain structure.^28^ This XRD results suggested that the presence of calcium ions and cysteine content showed significant impact on the interlayer spacing of bentonite.

**Figure 5:**
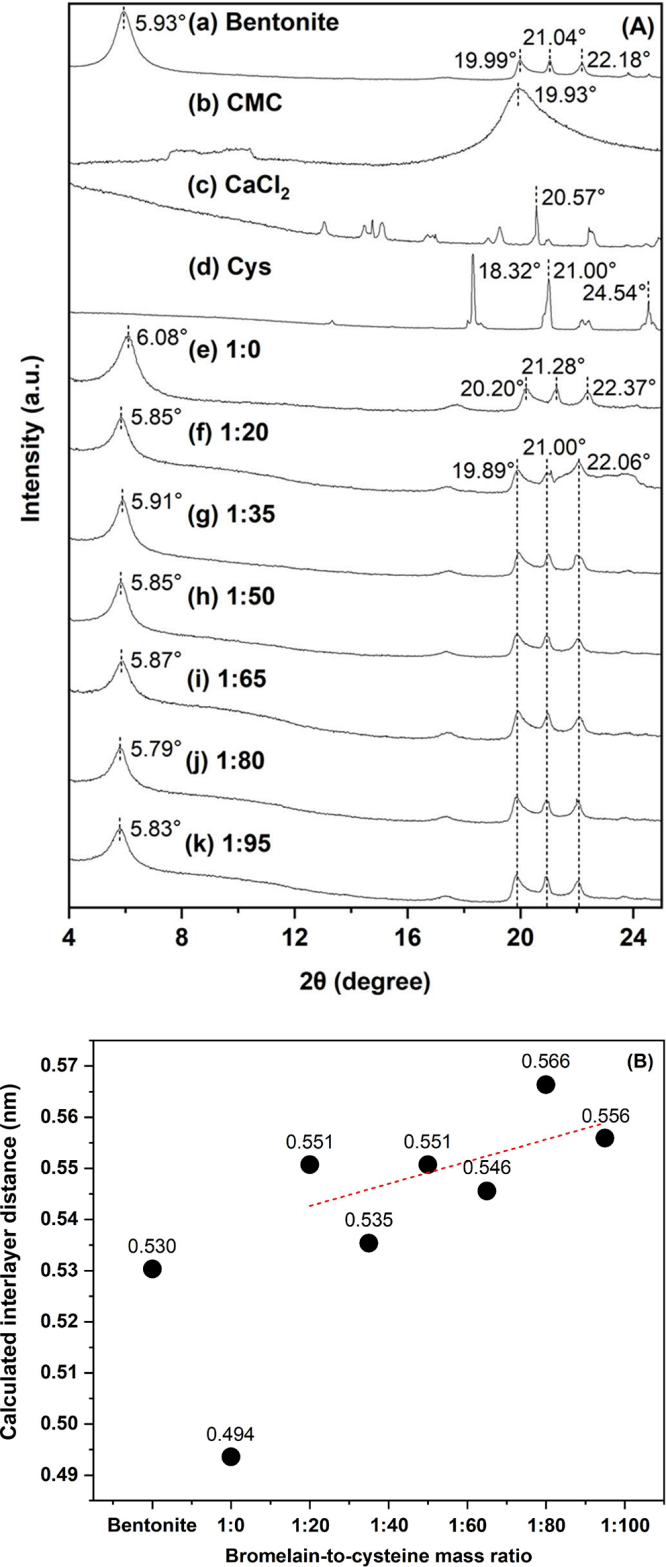
XRD profiles (A) of pristine Na-bentonite clay (a), CMC (b), calcium chloride (c), cysteine (Cys) (d), and CBr–Bt–CMC composites prepared at bromelain-to-cysteine mass ratios of 1:0 (e), 1:20 (f), 1:35 (g), 1:50 (h), 1:65 (i), 1:80 (j), and 1:95 (k) in a 2θ range of 4°–25°. Calculated interlayer distance (B) of bentonite and composites with different bromelain-to-cysteine mass ratios. The dashed line represents a trend of interlayer spacing presented in the composites with Br: Cys ratios ranging from 1:20 to 1:95.

### 3.3 Thermal Resistance of Free and Support-Bound Crude Enzyme

Thermal resistance of bromelain in crude solution and in composites with bromelain-to-cysteine mass ratios of 1:0 and 1:65 were investigated. The samples were incubated in 50 mM phosphate buffer (pH 7.0) under substrate-free conditions at 100 °C for 10 min, after which enzymatic activity was determined. They were then compared to their activity at 25 °C and expressed as residual activity, as shown in Figure 6. After treatment, the residual activity of free crude bromelain (CBr) decreased to almost 70%. This was caused by enzyme denaturation due to the oxidation of sulfhydryl groups in its active site.^53^ Meanwhile, immobilized bromelain with no addition of cysteine (1:0) exhibited less inactivation of the enzyme, maintaining nearly 100% of its residual activity. This was possibly caused by the presence of calcium ions in the composite environment that could altered enzyme conformation giving it enhanced properties such as resistance to irreversible thermal inactivation and a reversible active-inactive transition.^54^ After adding cysteine into composite (1:65), residual activity of the bromelain was obviously increased and higher than that obtained from the sample without cysteine (1:0) by almost 60%. This was likely caused by the presence of both calcium ions and cysteine molecules in the composites. Calcium ions could improve thermal stability while cysteine could act as a reducing agent to promote catalytic activity and to prevent active site in the immobilized bromelain from oxidation under thermal stress. In short, the composite demonstrates superior thermal stability with enhanced activity, showing its promising implementation in high-heat applications.

**Figure 6:**
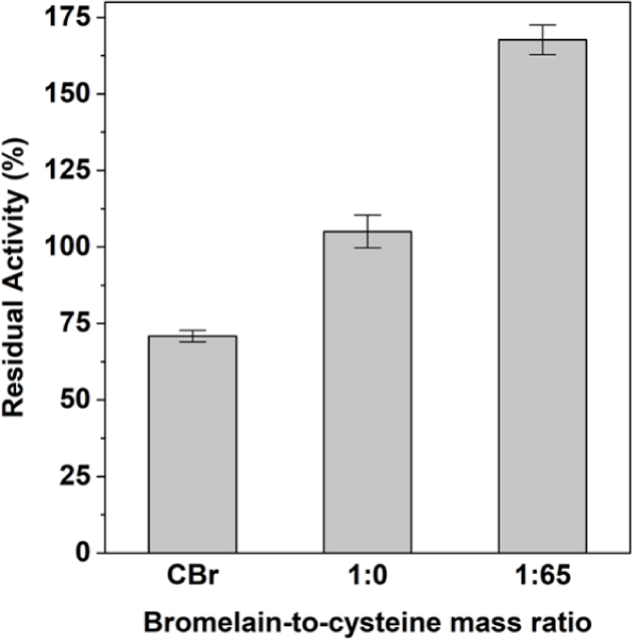
Residual activity of free crude bromelain (CBr) and immobilized CBr with bromelain-to-cysteine mass ratios of 1:0 and 1:65. Samples were treated at 100 °C for 10 min.

### 3.4 Influence of pH on Bromelain Catalytic Performance

Figure 7 depicts the influence of pH (3–10) on the catalytic activity of free and immobilized crude bromelain (CBr), using the composite prepared at a bromelain-to-cysteine mass ratio of 1:65. Free CBr retained relatively high activity (>90%) over a pH range of 6–10, whereas the immobilized enzyme exhibited comparable activity primarily between pH 4 and 6. Notably, the immobilized enzyme displayed better performance under acidic conditions compared to alkaline ones. This was possibly caused by the influence of positively charged calcium ions (Ca^2+^) and the presence of cysteine in solid support near active sites of bromelain.^55^ This resulted in the shift of a working pH range of enzyme to more acidic values, which was consistent with previous studies.^15,24^ A pronounced decrease in activity was observed at pH 3 for free bromelain and at pH 10 for the immobilized enzyme. This reduction is likely associated with weakened electrostatic interactions between the enzyme and substrate, leading to conformational alterations that limit catalytic flexibility.^56^ Compared with the free form, the shift in the pH corresponding to minimal activity for immobilized bromelain toward more alkaline conditions might be attributed to the combined influence of Ca^2+^ ions and cysteine presented within the support matrix.

**Figure 7:**
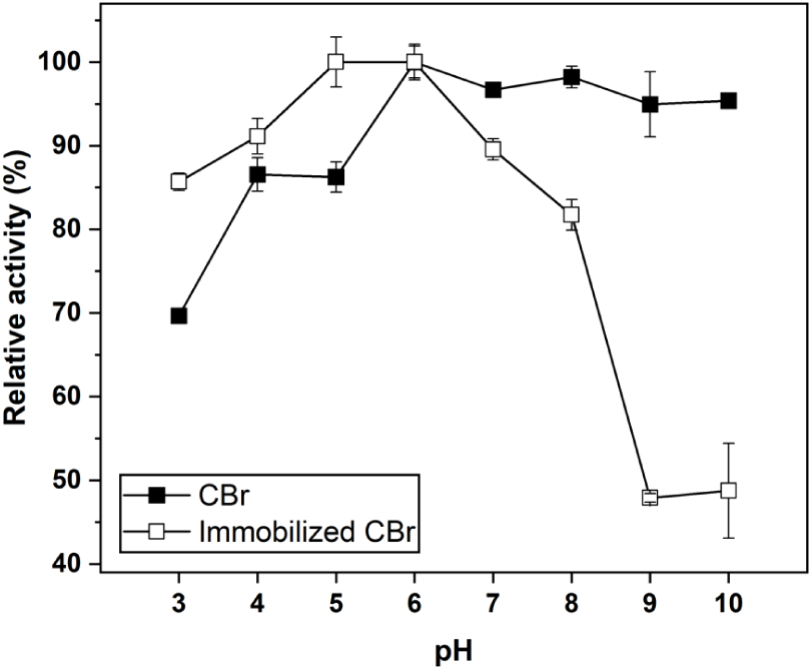
Influence of pH on catalytic activity of free and immobilized crude bromelain.

### 3.5 Stability under Storage Conditions

The storage stability of immobilized crude bromelain was evaluated by monitoring its relative activity over 10 days, as illustrated in Figure 8. Immobilized crude bromelain on Bt–CMC composites (bromelain-to-cysteine ratio 1:65) was employed in this experiment. To examine the effect of temperature and humidity on storage stability, the samples were stored in airtight containers and kept under 4 °C (10% humidity) and 25 °C (65–75% humidity) for comparison. After 2 days, enzyme activity at room temperature dropped sharply to approximately 30%, whereas the refrigerated sample retained around 70% of activity. This suggests that low temperature with low moisture could promote desiccation and reduce hydrolytic reactions such as autolysis in enzymes.^24,57,58^ Nevertheless, immobilized bromelain stored under both conditions retained approximately 28% of its initial activity after 10 days. When stored at 25 °C in vacuum-sealed, low-moisture packaging, the immobilized enzyme exhibited enhanced stability, maintaining around 50% residual activity on day 10. This improved storage stability is likely associated with the reduced moisture content in the storage environment.^24^ In summary, vacuum-sealed storage at 25 °C could be the most effective method in preserving enzyme activity because of its affordability and effectiveness in various industrial settings.

**Figure 8:**
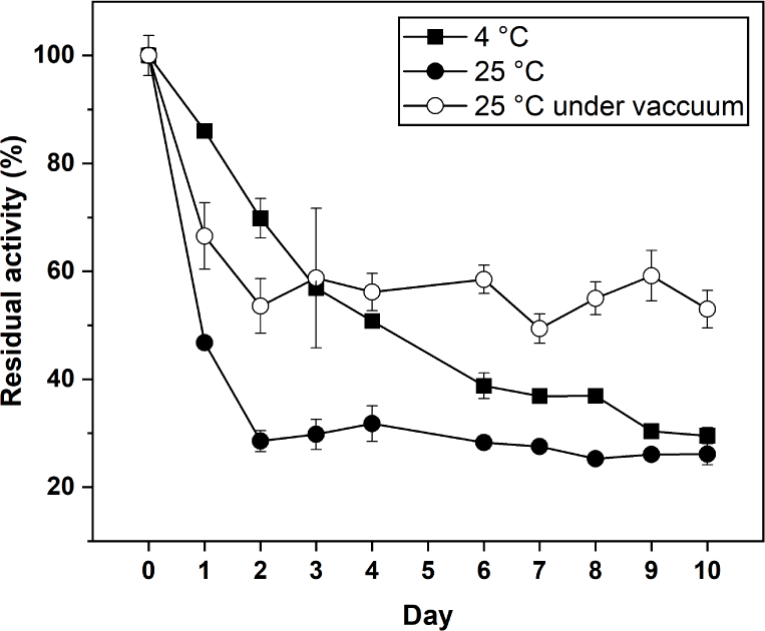
Storage stability of immobilized crude bromelain (CBr): comparison of storage in airtight containers at 4 and 25 °C, and in vacuum-sealed packaging at 25 °C.

### 3.6 Performance Evaluation of Immobilized Crude Bromelain in Soybean Meal Hydrolysis

The performance of immobilized CBr in soybean meal hydrolysis was assessed using the CBr–Bt–CMC composite with a bromelain-to-cysteine mass ratio of 1:65, selected based on its enhanced enzymatic activity and improved thermal stability. An appropriate mass ratio of SBM: composite for the hydrolysis reaction at 60 °C for 30 min was found at 1:4.5. (see Figure S1). Under these conditions, the nutritional value of SBM increased by approximately two- to 3-fold (16.48 ± 2.46 mg/L) compared with untreated SBM (7.68 ± 1.98 mg/L). The effects of incubation temperature (50, 60, 70, and 80 °C) and reaction time (15 and 30 min) on the hydrolysis process were further examined, as shown in Figure 9(A). The results indicate that the optimal temperature range was 60–70 °C, which is slightly higher than that reported in previous studies.^24^ Extending the hydrolysis time to 30 min resulted in approximately a 2-fold greater improvement in nutritional value compared with treatment for 15 min. Protein profiles of samples hydrolyzed at 60 and 70 °C for 30 min were further examined by SDS-PAGE, alongside untreated SBM and the CBr–Bt–CMC composite, as shown in Figure 9(B). The untreated SBM (lane 2) exhibited characteristic protein bands corresponding to the α′, α, and β subunits of β-conglycinin (78, 70, and 47 kDa) as well as the acidic and basic subunits of glycinin (37 and 19 kDa).^59,60^ These proteins are recognized as the major allergenic components in SBM.^61^ For the component released from the composite (lane 3), a distinct band with a molecular weight below 10 kDa was observed, which can be attributed to cysteine added during composite preparation. Following enzymatic treatment, electrophoretic profiles of all treated SBM samples (lanes 4 and 5) predominantly exhibited protein bands below 10 kDa, indicating extensive protein hydrolysis and the apparent absence of major allergenic protein bands. The molecular mass distribution of peptides generated from these samples was further analyzed by MALDI-TOF mass spectrometry. In untreated SBM, characteristic signals at 11,306 *m*/*z*, 15,225 *m*/*z*, 20,594 *m*/*z*, and 25,487 *m*/*z* were detected (see Figure S2). In contrast, no detectable protein signals were observed in the mass range of 3,000–12,000 *m*/*z* for any treated SBM samples (see Figure S3). These results suggest that soybean proteins were efficiently hydrolyzed into low-molecular-weight peptides, predominantly below 3,000 *m*/*z* (≈3 kDa), within 30 min of treatment. This low molecular weight and less complex structure of the peptides enabled them to be easily absorbed and bioavailable to their intended biological destinations.^62–64^ Overall, these findings indicate that the CBr–Bt–CMC composite is effective in promoting soybean protein hydrolysis, enhancing nutritional quality, and potentially reducing allergenic protein content in SBM.

**Figure 9:**
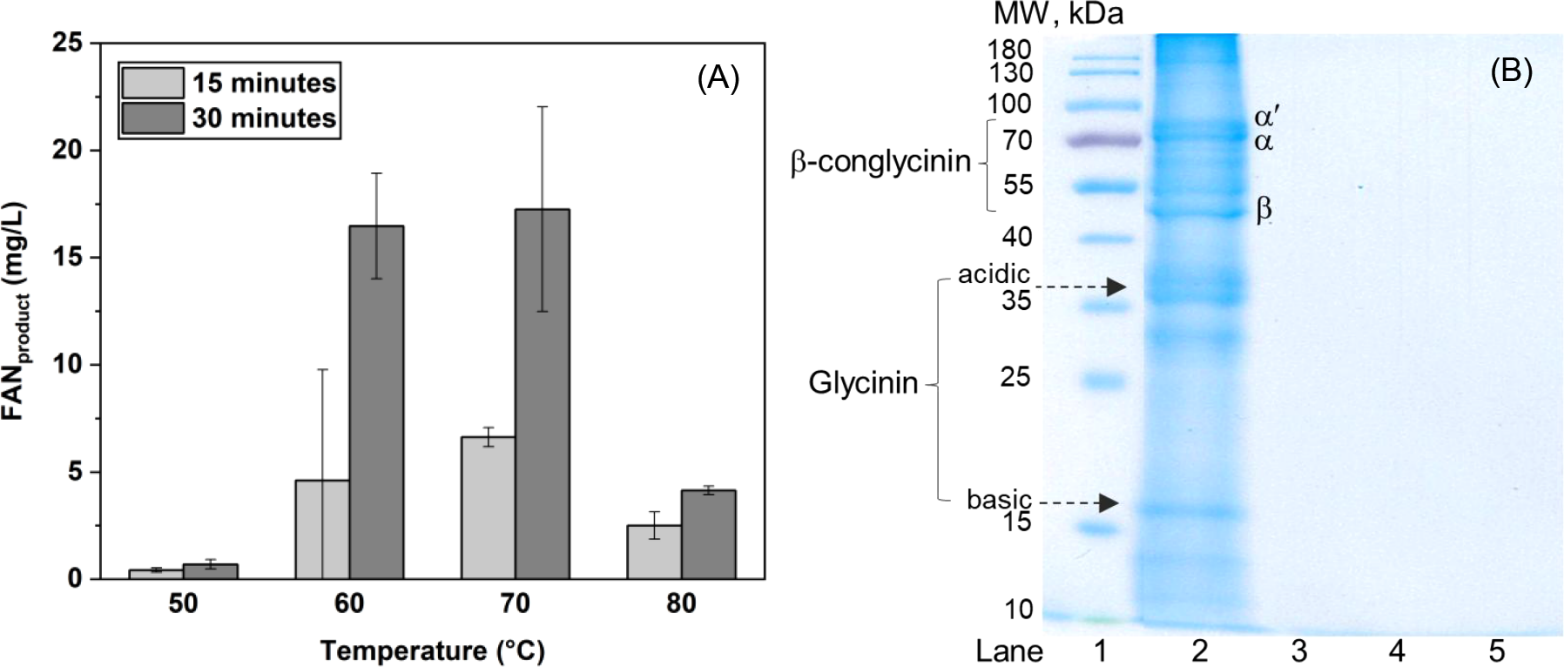
(A) Free alpha-amino nitrogen as a product $\left(FAN_{product}\right)$ of SBM hydrolysis by composite at different temperatures (50, 60, 70, and 80 °C) and reaction times (15 and 30 min). (B) SDS–PAGE analysis showing the hydrolytic performance of immobilized crude bromelain toward soybean meal (SBM): molecular weight markers (lane 1), untreated SBM protein subunits (lane 2), CBr–Bt–CMC composite (lane 3), and SBM hydrolyzed with immobilized bromelain at 60 °C (lane 4) and 70 °C (lane 5) for 30 min.

The reusability of immobilized crude bromelain in soybean meal hydrolysis was evaluated over four consecutive cycles at 60 °C using incubation times of 15 and 30 min (Figure 10). Across all cycles, the 30 min treatment produced approximately 2–4 times higher free amino nitrogen (FAN) than the 15 min condition, indicating higher single-batch hydrolysis efficiency. However, residual activity under the 30 min condition decreased sharply after the first cycle and stabilized at ∼60–70% in subsequent cycles, whereas the 15 min treatment maintained near-complete activity through three cycles before declining to ∼60% in the fourth cycle. This indicates that shorter incubation helps preserve structural integrity and prolongs the functional lifespan of the immobilized enzyme.^65^ Overall, the immobilized enzyme retained over ∼60% of its initial activity after four cycles, demonstrating good operational stability of the immobilized enzyme and highlighting a trade-off between hydrolysis efficiency and long-term reusability depending on application requirements. These results confirm that the immobilized bromelain system is suitable for repeated use under process-relevant conditions.

**Figure 10:**
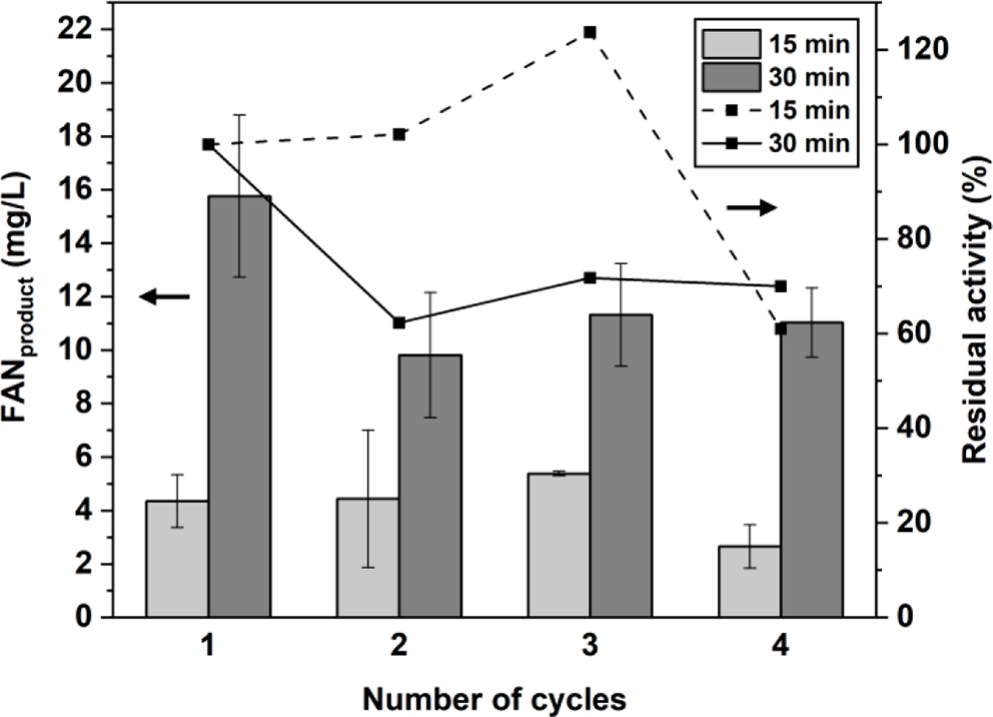
Reusability of immobilized crude bromelain (CBr–Bt–CMC composite) in successive soybean meal hydrolysis cycles. Free amino nitrogen (FAN) production after 15 and 30 min of reaction (left axis) and residual enzymatic activity (right axis) are shown over four consecutive cycles.

## 4 Conclusions

Crude bromelain (CBr), extracted from pineapple peel waste, was successfully immobilized onto a bentonite-carboxymethylcellulose (Bt–CMC) composite with the addition of cysteine, using ionotropic gelation technique. The resulting CBr–Bt–CMC composite achieved an immobilization yield exceeding 80%. It was obviously found that the addition of cysteine during composite preparation process could significantly improve catalytic activity of the immobilized CBr. Notably, the composite prepared with a bromelain-to-cysteine mass ratio of 1:65 demonstrated a remarkable enhancement in catalytic activity over 1,000% compared to the composite without cysteine (1:0). It also retained 167.68 ± 4.86% of its initial activity after 10 min of heat treatment at 100 °C, indicating excellent thermal stability. X-ray diffraction (XRD) analysis exhibited that the addition of cysteine, in the presence of calcium ions, led to an expansion of the bentonite interlayer spacing. The shift of a working pH range of CBr to more acidic values was also observed after immobilization, which was influenced by alterations in the local charge environment near the enzyme’s active sites. The application of immobilized CBr for soybean meal (SBM) hydrolysis was also evaluated. The composite effectively degraded SBM proteins at 60–70 °C within 30 min, resulting in a 3-fold increase in nutritional value. Furthermore, it facilitated the breakdown of two major allergenic proteins in SBM, including glycinin and β-conglycinin, and generated low-molecular-weight peptides (less than 3 kDa) enabling bioavailability improvement. The system also exhibited measurable reusability, with approximately 60% of the initial activity retained after four consecutive hydrolysis cycles, highlighting its potential for repeated use. While this study demonstrates effective immobilization and application of crude bromelain derived from pineapple peel waste with promising stability, reusability, and catalytic performance, further investigation into activity-based immobilization metrics and long-term continuous operation would provide deeper mechanistic insight and support broader industrial implementation.

## Supplementary Material



## Notes

Details regarding free alpha-amino nitrogen as a product (FAN_product_) of SBM hydrolysis by mixing the CBr–Bt–CMC composite with various SBM-to-composite mass ratios (1:0.1, 1:0.5, 1:1, 1:2.5, 1:4.5, 1:7, and 1:10); mass spectrometry analysis of treated SBM with immobilized crude bromelain at 60–70 °C for 30 min; and measurements were performed in linear positive-ion mode over mass ranges of 3,000–20,000 m/z and 10,000–200,000 m/z (PDF)
