# Supplementary material for: Valorization of Pineapple Peel Waste through Immobilized Crude Bromelain for Enhanced Feed Protein Hydrolysis
Source: ACS Omega. 2026 Jan 21;11(4):5468–78. doi: 10.1021/acsomega.5c08847 (PMC12878712; doi:10.1021/acsomega.5c08847)
Supplement: Supplementary file 1 [file ao5c08847_si_001.pdf]

Supporting Information

Valorization of Pineapple Peel Waste through Immobilized Crude Bromelain for Enhanced Feed Protein Hydrolysis

Chanyakan Skulborisutsuk[a], Maythee Saisriyoot[b], Songwut Suramitr[c], Yodying Yingchutrakul[d], Chutikarn Butkinaree[d], Ryuichi Egashira[e], Lapporn Vayachuta[f], Panida Prompinit[f,] *

[a]Interdisciplinary of Sustainable Energy and Resources Engineering, Faculty of Engineering, Kasetsart University, Bangkok, 10900, Thailand
[b]Department of Chemical Engineering, Faculty of Engineering, Kasetsart University, Bangkok, 10900, Thailand
[c]Department of Chemistry, Faculty of Science, Kasetsart University, Bangkok, 10900, Thailand
[d]National Center for Genetic Engineering and Biotechnology, National Science and Technology Development Agency (NSTDA), Pathum Thani, 12120, Thailand
[e]Department of Transdisciplinary Science and Engineering, School of Environment and Society, Institute of Science Tokyo, Tokyo, 152-8550, Japan
[f]National Nanotechnology Center (NANOTEC), National Science and Technology Development Agency (NSTDA), Pathum Thani, 12120, Thailand

Correspondence
Panida Prompinit, National Nanotechnology Center (NANOTEC), National Science and Technology Development Agency (NSTDA), Khlong Luang, Pathum Thani, 12120, Thailand
Email: panida@nanotec.or.th

1. Performance evaluation of the immobilized crude bromelain in soybean meal hydrolysis

Experiment

Performance of the immobilized crude bromelain was evaluated by considering hydrolysis of soluble protein in soybean meal (SBM). Firstly, 0.1 g of SBM powder (<250 μm of particle size) was mixed with the composite at different SBM-to-composite mass ratios of 1:0.1, 1:0.5, 1:1, 1:2.5, 1:4.5, 1:7, and 1:10. For each sample, 6 mL of deionized water was added to the mixture, followed by an incubation at 60 ºC for 30 min in a shaking water bath with a speed of 120 rpm. After the incubation period, the mixture was boiled at 95 ºC for 30 min, followed by addition of 4 mL of deionized water. The mixture was centrifuged at 8000g for 10 min at 4 ºC. The supernatant was collected for further free alpha-amino nitrogen (FAN) analysis using Ninhydrin method. In summary, 1 mL of the supernatant was combined with 3 mL of deionized water, followed by addition of 1 mL of 2% Ninhydrin solution prepared in ethanol. The mixture was then shaken and heated at 95 ºC for 15 min. After cooling for 20 minutes, 1 mL of 50% ethanol was added. The absorbance at 570 nm was measured and the free amino acid (FAN) content was calculated with a glycine standard curve. Calculations were carried out using Equation (1),

$$FAN\ (mg/L) = \frac{A_S - A_B}{A_G - A_B} \times 2 \times F \quad (1)$$

where, A_S is an average absorbance of sample, A_B is an average absorbance of a blank value, A_G is the average absorbance of glycine standard solution, 2 is the concentration of glycine standard solution in mg/L, and F is a sample dilution factor. The free amino acid (FAN) contents were determined in composite ($FAN_{composite}$), SBM (FAN_{SBM}), and the mixture between the composite and SBM after treatment period ($FAN_{composite\,\&\,SBM}$). FAN product released from SBM hydrolysis ($FAN_{product}$) by the immobilized enzyme was calculated using Equation (2),

$$FAN_{product}\ (mg/L) = FAN_{composite\,\&\,SBM} - FAN_{composite} - FAN_{SBM}. \quad (2)$$

Results

Performance of the immobilized bromelain in soybean meal hydrolysis was further evaluated by using the CBr-Bt-CMC composite with a bromelain-to-cysteine mass ratio of 1:65 due to its significant improvement in enzyme activity and thermal stability. To optimize the amount of the composite, the hydrolysis reaction was performed at 60 °C for 30 min with various SBM-to-composite mass ratios of 1:0.1, 1:0.5, 1:1, 1:2.5, 1:4.5, 1:7, and 1:10. The free amino acid (FAN) contents were determined in composite ($FAN_{composite}$), SBM (FAN_{SBM}), and the mixture between the composite and SBM after treatment period ($FAN_{composite\,\&\,SBM}$). FAN product released from SBM hydrolysis ($FAN_{product}$) by the immobilized enzyme was calculated using Equation (2). The results were shown in **Figure S1**. It was found that SBM hydrolysis occurred when the SBM-to-composite mass ratio exceeded 1:1. The $FAN_{product}$ increased as the SBM-to-composite mass ratio increased and reached 16.48 ± 2.46 mg/L at a ratio of 1:4.5. This nutritional value was increased by ~2−3 times from that presented in untreated SBM (7.68 ± 1.98 mg/L).

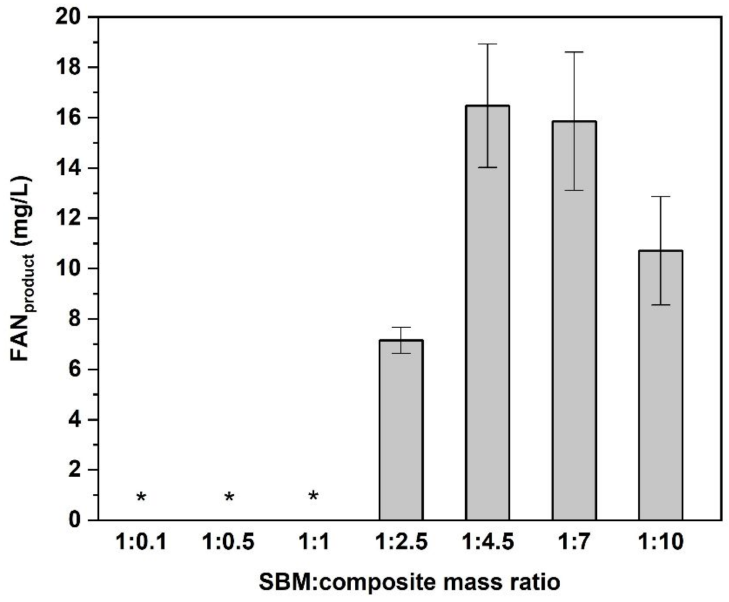

Figure S1. Free alpha-amino nitrogen as a product ($FAN_{product}$) of SBM hydrolysis by mixing the CBr-Bt-CMC composite with various SBM-to-composite mass ratios (1:0.1, 1:0.5, 1:1, 1:2.5, 1:4.5, 1:7, and 1:10). An asterisk denotes an undetectable FAN product. All experiments were performed under a fixed condition of 60 °C for 30 min.

2. Mass spectrometry analysis

Low molecular mass distributions of untreated SBM, CBr-Bt-CMC composite, and SBM after treatment with the composites at 60 and 70 ºC for 30 min were analyzed using mass spectrometry technique or MALDI-TOF MS analysis. 5 µL of each desalted sample was mixed with the MALDI matrix solution in a ratio of 1:1 (sample to matrix). The mixture was spotted onto the 384-spot MALDI target plate (JEOL, Japan) and left to dry at 25 °C. The matrix solution was 10 mg sinapic acid (SA) in 50% acetonitrile containing 0.1% trifluoroacetic acid (TFA). Subsequently, the MALDI target plate was loaded into the JMS S-3000 spiralTOF (JEOL, Japan). The mass spectra were acquired with the following conditions: 57% laser energy, linear mode, positive ionization, target plate 20 kV, 250 Hz laser frequency, and 6,250 laser shots.

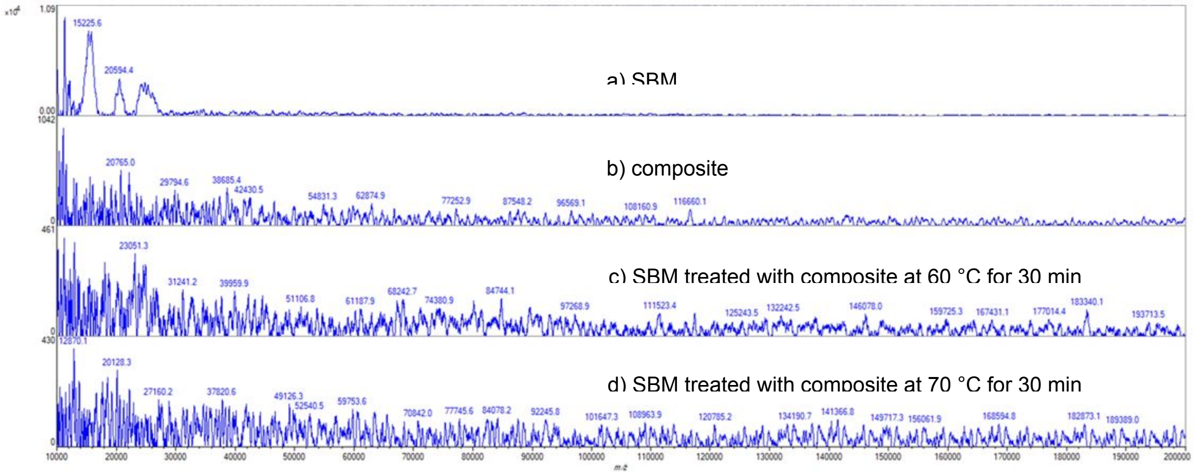

Figure S2. Mass spectra of soybean meal (SBM), composite, and SBM after treatments with immobilized bromelain at 60 and 70 °C for 30 min. The measurements were carried out in a linear mode, positive ionization, mass range: 10,000-200,000 *m/z*.

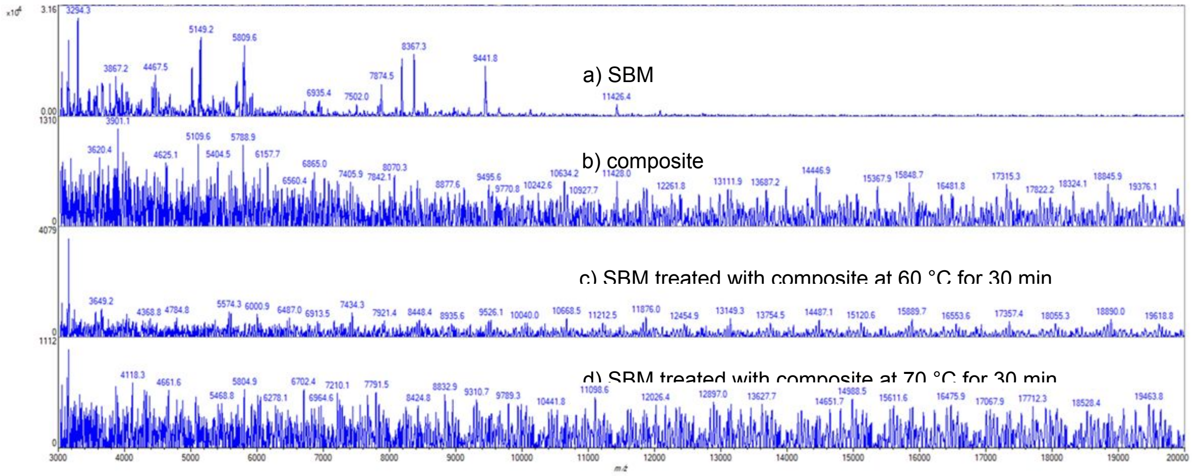

Figure S3. Mass spectra of soybean meal (SBM), composite, and SBM after treatments with immobilized bromelain at 60 and 70 °C for 30 min. The measurements were carried out in a linear mode, positive ionization, mass range: 3,000-20,000 *m/z*.
